# Information work and digital support during the perinatal period: Perspectives of mothers and healthcare professionals

**DOI:** 10.1371/journal.pdig.0000387

**Published:** 2024-08-16

**Authors:** Emma Kemp, Elizabeth Sillence, Lisa Thomas

**Affiliations:** 1 School of Psychology, Sheffield University, Sheffield, United Kingdom; 2 Department of Psychology, Northumbria University, Newcastle upon Tyne, United Kingdom; Iran University of Medical Sciences, ISLAMIC REPUBLIC OF IRAN

## Abstract

During pregnancy and early motherhood, the perinatal period, women use a variety of resources including digital resources to support social interactions, information seeking and health monitoring. While previous studies have investigated specific timepoints, this study takes a more holistic approach to understand how information needs and resources change over the perinatal period. Furthermore, we include the perspective of maternity healthcare professionals to better understand the relationship between different stakeholders in the information work of perinatal women. A total of 25 interviews with 10 UK based mothers and 5 healthcare professionals (3 Midwives and 2 Health visitors) were conducted. Perinatal women were asked about their information and support needs throughout pregnancy and the postnatal period, healthcare professionals were asked about information and support provision to perinatal women. Information work activities were grouped along stages of the perinatal timeline from pre-pregnancy to the postanal period to illustrate the work and perspectives of the women and the healthcare professionals. Information work varies considerably over the timeline of the perinatal period, shifting back and forth in focus between mother and baby. information work during this period consists of many information related activities including seeking, monitoring, recording, questioning, sharing and checking. The importance of the HCPs as stakeholders in this work is notable as is the digital support for information work. Importantly, paper-based resources are still an important shared resource allowing reflection and supporting communication. Information work for women varies across the perinatal timeline. Particular challenges exist at key transition points, and we suggest design considerations for more integrated digital resources that support information work focused on mother and baby to enhance communication between perinatal women and healthcare professionals.

## 1. Introduction

During pregnancy and early motherhood, mobile health apps and social media can be beneficial tools for social support, education, information seeking and health monitoring [1–3]. Online health information seeking plays an important role during this time and pregnant women enjoy monitoring their own health along with fetal growth during the different stages of pregnancy [4]often using mobile apps to access information about specific pregnancy symptoms, nutrition, antenatal tests and labour and birth [5]. For pregnant women, digital resources supplement (insufficient) information provided during early prenatal visits with health care professionals (HCPs) [6]. Although, it is not always clear that the information sought and retrieved from online sources is shared with HCPs [7].

The transition from pregnancy to motherhood can prompt many women to seek information on topics such as breastfeeding, sleep schedules, post-natal self-care, and mental health with the information focus switching between baby and mother. Research has shown that first time mothers require emotional support from health professionals alongside physical checks and written information [8]. A study of first- time mothers’ expectations of postnatal care, for example, revealed that new mothers would prefer physical and mental health checks to be part of their postnatal care however in reality only checks on mother and baby’s physical health occurred [9]. The sudden shift in role and the responsibility of caring for a newborn can leave some mothers feeling overwhelmed and in need of both social and informational support. In the UK, NICE guidelines [10] 2021, policy states that the standard structure of postnatal care (excluding high risk women who are offered further postnatal care) involves a midwife home visit within the first 36 hours from the transfer of care from place of birth to home. A health visitor home visit is arranged between seven to fourteen days after transfer of care, and an additional GP appointment is made for six to eight weeks postnatal involving a physical examination of both mother and baby [10]. Interactions with HCPs at these transition points often see an end to continuity of care with some relationships ending and new ones beginning, and this has consequences for the ways in which information is sought, shared and stored, in other terms it impacts upon the information work that is taking place.

### 1.1 Information work

The notion of information work coined by Corbin and Strauss [11] explores how people make sense of health information to enable them to manage chronic illness in their everyday lives. The term was further conceptualised by Dalmer & Huvlia [12] and Mazanderani et.al [13] to show how people engage with health information including how the information is found and managed by individuals to enable them to manage their own health conditions. The term ‘information work’ is a useful way of considering how people interact and engage with information and provides a lens through which we can examine and acknowledge the different resources, functions and stakeholders related to information activities. The increasing emphasis on the role of individuals in managing their own health is particularly relevant in the context of digital resources. Though digital support can be beneficial to meet the information needs of expectant and new mothers, [6], online information can be poor quality and many health apps can be unreliable and contain inappropriate information [14] adding to the information work burden.

In this study we use the concept of information work to examine changing information practices (online and offline) across the perinatal timeline. We conducted a thematic analysis of 25 interviews with mothers and healthcare professionals (HCPs) collected in the UK between 2019 and 2021 to explore information seeking, storing and sharing needs and practices and to examine tools and resources to support information work (digital and non-digital). By examining the perspectives of perinatal women and HCPs we seek a more holistic account of needs and provision across the pregnancy timeline. We find that information work is ongoing and effortful despite the offline and online resources available. The use of digital support for information work varies across the timeline from early pregnancy to postnatal, with paper-based resources often being used and valued during this time to assist with information provision and exchange.

In this paper, we make the following contributions to the literature.

1. We describe how the information needs; resources and stakeholders change over the perinatal timeline. While previous work has examined individual timepoints from early pregnancy through to birth and beyond, we take a more holistic approach covering the entire timeline to include the postnatal period.
2. We include the perspective of maternity healthcare professionals to better understand the relationship between different stakeholders in the information work of perinatal women.
3. We present design considerations for digital tools to support perinatal women across pregnancy and during the post-natal period.

Below we present a brief overview of the literature on social and information support for mothers and the role of digital resources in this context. We also introduce the current UK maternity healthcare context and summarise work on HCP perspectives. Next, we describe our methods followed by results which are structured using a perinatal timeline approach to highlight the changing dynamics of the information work from pre/early pregnancy to the postnatal period.

## 2. Background

### 2.1. Social and informational support for maternal wellbeing

Throughout pregnancy and beyond, women’s social and informational needs change. A key source of information throughout pregnancy remains the midwife [15]. In the UK, the role of the midwife is to provide professional support and care to pregnant women during pregnancy. The role involves providing evidence-based information and helping women to make informed choices about their options and services available throughout pregnancy [16] After birth, a health visitor (a nurse or midwife with additional specialist training) continues to provide support for the baby and family and is the point of contact until the child goes to school [16]. Women regard ‘discussion with midwife’ as the greatest source of information throughout pregnancy [15,17] and as such, face to face antenatal appointments remain an important opportunity for information exchange during pregnancy.

In addition to professional support, providing new mothers with access to a supportive community to help cope with the transitions from pregnancy to motherhood and the challenges that come with caring for a newborn is essential. Social support increases maternal health, child development and coping as a new mother [18]. Social support is often sought and found in close family and friends. Seeking support from partners can be advantageous to mothers’ mental health [19], and mothers who have partners they perceive as ‘available to offer help’ report reduced symptoms of depression, anxiety and parental burnout. Partners who offer support during labour often continue this support into the postnatal period [20] which is valuable to mothers’ postnatal mental health.

### 2.2 Technology use in pregnancy and beyond

Online social support, through social media, online forums and mobile apps can provide pregnant women and new mothers with the opportunity to be part of a community and share experiential information related to pregnancy and the postnatal period. Information sharing online can lead to feelings of empowerment amongst pregnant women, who feel more in control over their changing bodies and know how to prepare for their child [4]. First time mothers can benefit from online social networking by connecting with other new mothers to access information and support [21]. In addition, using online sources of information and support can have positive effects on mental health including reduced anxiety [5].

During the early pregnancy phase (first trimester) pregnant women have highlighted a gap in care where a lack of information and contact occurs with healthcare professionals (HCPs) and they often use technology platforms to ensure their information needs are met during this stage [6]. Postnatal mothers experience a further gap in care, where provision of information and support is reduced. Women often experience a greater focus on the health and wellbeing on their baby rather than on themselves during postnatal HCPs appointments [8] and this is referred to as the ‘invisible mother’ [22]. The first six weeks of the postnatal period have been identified as a period where new mothers desire increased information and support from HCPs [23] but if this is not forthcoming then new mothers turn to other sources of information to meet their needs during this stage [8].

In the UK, the NHS has attempted to embrace the role of digital technology in supporting pregnant and postnatal women alongside their HCPs. The NHS currently promotes the app Baby Buddy to expectant mothers, which contains information about maternal and foetal health during pregnancy up to six months postpartum. The app contains an ‘Ask me’ feature which aims to answer pregnancy related questions with digitally stored expert information. Evidently, technology can play a role in filling information gaps and providing support at different times through pregnancy and postnatally.

### 2.3 The role of healthcare professionals

Despite HCPs being a widely used information source, women also face barriers to accessing this source of information and having their information needs met through pregnancy. Problems women face include a reluctance to discuss personal pregnancy related issues with HCPs due to feeling ashamed or embarrassed, lack of communication with HCPs, reliance on self to seek information, lack of awareness of appropriate information sources and inadequate information provided by healthcare professionals throughout pregnancy [24]. It is evident that HCPs play an important role in facilitating information seeking for new mothers however, it is apparent that relationships between women and maternity healthcare providers affects how success or otherwise of the exchange of information [25]. Women have shown reluctance to share personal information and receive information if the foundation of a trusting relationship has not been established. Mothers have rated HCPs as their main source of trusted information; however, family was the most common information source used [26] showing that mothers rely on health professionals to provide trustworthy information but may face barriers (such as gaps between appointments) to accessing this source of information consistently throughout the perinatal period.

### 2.4 Rationale

Research has shown that during pregnancy and early motherhood, technology can be beneficial for social support, education, information seeking and health monitoring. However, most evidence appears to focus on a specific time of pregnancy and postpartum, therefore understanding how information seeking, relationships with HCPs and technology interactions change over this time frame is difficult. This study therefore aims to understand perinatal mothers’ perspectives of specific technology use as well as interactions with health services over the perinatal period. There is also limited research examining the perspective of health professionals across this time frame, therefore the views of professionals with experience of delivering care to perinatal mothers will be examined to understand more about the information exchange opportunities that occur as part of their role.

### 2.5 Research question

To address the gaps in literature, the following research questions were identified:

1. How does information work occur through the perinatal period and what role does technology play in facilitating relationships or meeting information needs of expectant and new mothers?
2. How is support and information provided to perinatal mothers from a health professional perspective?

## 3. Method

### 3.1. Participants

A total of 15 participants were recruited. This included 10 UK based mothers who were either pregnant or had given birth in the previous twelve months and 5 maternity healthcare professionals. (3 midwives, 2 Health visitors).

In addition, two of the pregnant participants (P2 and P3) volunteered to take part in a series of follow up interviews postnatally at monthly intervals until four months after the birth of their child and then again at six months postnatally. This resulted in a total of 25 interviews. The sample represented a population of perinatal women who mostly resided in Northeast England, UK (N = 9) and maternity HCPs who practiced mostly in Northeast England (N = 3) (see Tables 1 and 2 for full demographic details) Purposive sampling methods were used to recruit participants via social media and personal networks of peers and healthcare professionals. The researchers approached either met potential participants and invited them to participate, providing them with oral information and a participant information sheet or potential participants were asked to contact the researcher EK via email to take part if recruited via social media. Inclusion criteria for perinatal women to take part in the study was, aged over 18, living in the UK and either currently pregnant or had given birth in the previous 12 months. Inclusion criteria for HCPs was to be a current or previously practicing trainee or qualified midwife or health visitor in the UK. Both current practising and retired HCPs were included in the sample to assess how the provision of antenatal and postnatal care has changed over time and how retired HCPs perceive the current practice. This offered further insight into how the structure of care has changed and how this might impact new mothers, particularly around the reduction of HCP contact in the postnatal phase.

**Table 1:** *Participant demographics for perinatal women*.

Participant no/Pseudonym	Stage of gestation	Child no.	Race/Ethnicity	Location	Age
**1.Sarah**	Third trimester	First child	White British	Newcastle	25–30
* **2. Rachel**	Third trimester	First child	White British	Northumberland	25–30
* **3. Claire**	Second trimester	First child	White British	Northumberland	35–40
**4. Isobel**	Second trimester	Second child	White British	London	30–35
**5. Alice**	6 months postnatal	Fourth child	White British	Northumberland	25–30
**6. Amy**	Third trimester	First child	White British	Northumberland	30–35
**7. Sam**	4 months postnatal	First child	White British	Newcastle	30–35
**8. Lauren**	10 months postnatal	First child	White British	Northumberland	25–30
**9. Nicola**	12 months postnatal	Second child	White British	Northumberland	25–30
**10. Chelsey**	1 month postnatal	First child	White British	Newcastle	25–30

* Participant took part in follow up interviews at 1, 2, 3, 4, 6 months postnatally.

First trimester- conception to 12 weeks gestation. Second trimester, 13–27 weeks gestation. Third trimester, 28–40-week gestation. Postnatal, post-birth to 12 months.

**Table 2:** *Participant demographics for HCPs*.

Profession/ Pseudonym	Occupation status	Location	Race/ethnicity	Experience
**Midwife (Lindsey)**	Currently practising	Northeast England	White British	Newly qualified (less than 2 years)
**Midwife (Carol)**	Currently practising	Lancashire, England	White British	10+ years
**Midwife (Susan)**	Retired	Northeast England	White British	20+ years
**Health Visitor (Catherine)**	Currently Practising	Northeast England	White British	10+ years
**Health Visitor (Kate)**	Retired	South Yorkshire, England	White British	10+ years

#### Interviews

For the main interviews for perinatal women and HCPs, a semi-structured interview schedule was devised to cover the pregnancy and postnatal timeline but was flexible to explore the individual circumstances (such as being a first- or second-time mother) of each participant. Interview schedules for HCPs were tailored to suit the midwife and health visitor roles. Topics included: (1) pre-pregnancy information seeking and technology use, (2) information needs and provision of information, (3) patterns of information exchange between women and HCPs, (4) relationships between women and HCPs, (5) technology use along the timeline and (6) opinions of developing an app to improve communication between women and HCPs. Interview questions relating specifically to the midwives included ‘Can you tell me about the general timeline to your meetings antenatal meetings?’, ‘How would you say your relationship with your client develops over the course of pregnancy?’, and those specific to health visitors included ‘What information do you provide to a new mum during the first postpartum visit?’, ‘What kind of support do you/can you offer to a new mum who was struggling with postpartum mental health or physical recovery’.

#### Follow up interviews

**Fig 1:**
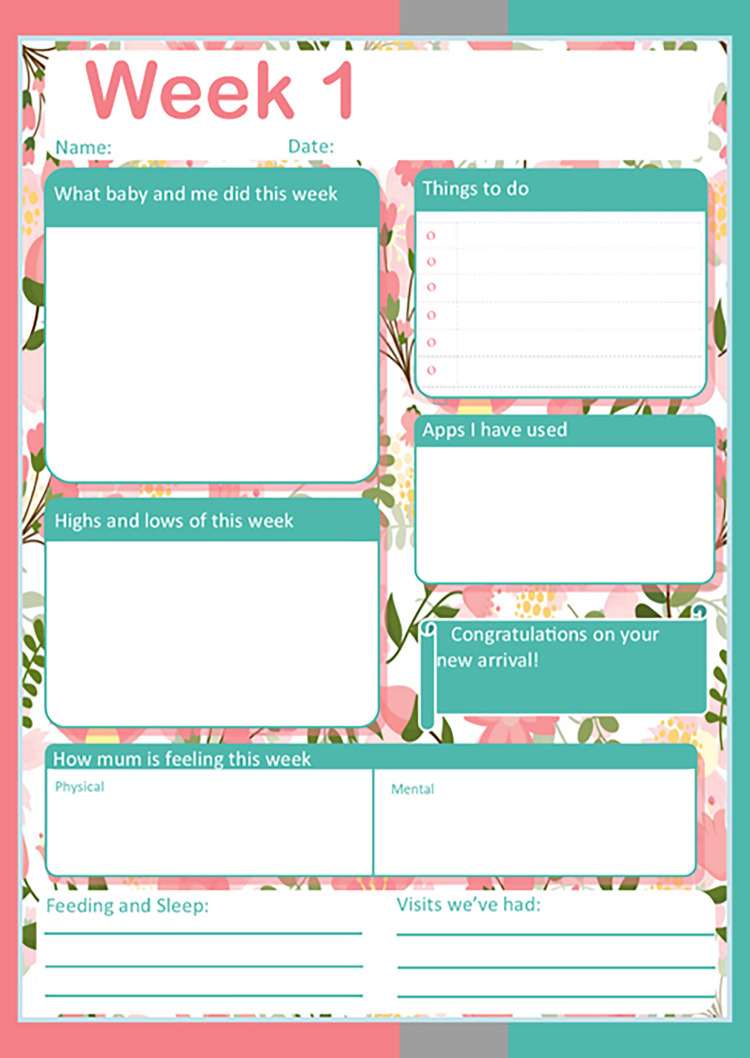
‘New mum’ journal.

For the follow up interviews a weekly ‘New mum journal’ spanning birth to twelve weeks postnatal, was created to allow new mothers to record personal data about themselves or infant. The new mum journal (see Fig 1) was used to capture information about digital and non-digital sources used as well as information about mother and baby that could then be used as a prompt during interviews. The design of the journal allowed new mothers to focus and reflect on their own physical and mental wellbeing following birth, while also allocating time to record baby’s progress in these initial stages. The journal was designed to appear bright and user friendly, with clear sections and weekly positive messages/milestones such as ‘congratulations’ or reminders ‘6-week check-up due’ as prompts to new mothers. Wording in the journal was designed to be informal and engaging to appeal to the audience. Sections were designed so mothers could bullet point information or write short extracts. A ‘notes’ section was included on the back page to each weekly page to allow mothers to make additional notes or include photographs. Progress could be tracked over the initial twelve weeks postnatal and personal information stored in the journal could be kept as a record to present to HCPs should any concerns regarding mother and baby arise.

Both participants were interviewed at months 1,2,3,4, and 6 postnatal to discuss both their thoughts on the ‘new mum journal’ and what information and support were sought and received during the initial 6 months on motherhood. From months 1 to 3, participants were prompted to fill in their journals and bring the journal to the interview to discuss. Interview topics covered information seeking and sharing, HCP communication and changing concerns about self and baby. The interview schedule was adapted throughout the six-month period to reflect the changing information needs and sources used during this time (for example having less contact with HCPs after three months postnatal and discussion of greater use of technology to support information needs).

### 3.2 Procedure

Interviews took place either face to face (at the participant’s home or a quiet location at the University) or remotely depending on preference. All the HCPs and one of the perinatal women were interviewed via FaceTime. All the main interviews lasted between 30 minutes to 1 hour and all follow up interviews were conducted at the participant’s home and lasted between 40 minutes and 1 hour.

### 3.3 Ethics statement

The Northumbria University Ethics Committee (submission reference 4495) approved the study. Written formal consent was gained from all participants prior to data collection commencing. All participants were provided with a participant information sheet and consent form. A debrief form was given to participants immediately following the interviews. This reiterated the nature of the study and provided participants with details of how their data would be stored and how to withdraw their data from the study if necessary. Consent was gained from each participant prior to the interview commencing. To ensure GDPR regulations were met, pseudonyms were used to maintain the anonymity of all participants. All data was stored securely on password protected cloud-based university storage.

### 3.4 Data collection and analysis

All interviews were recorded using the ‘Easy Voice Recorder’ mobile app and transcribed into written text. The first author used inductive thematic analysis [27] to analyse the data supported by the NVivo software program. While analysing the data, (10 perinatal women, and follow-up interviews) it was evident that the information work of new mothers varies across the timespan from early pregnancy to postnatal. Therefore, developing codes were grouped by time point along the perinatal period which were presented as themes. At each time point, key information needs, activities and practices were identified and coded. To ensure a rigorous analysis process, author EK performed the initial coding and grouping into themes and 10% of transcripts were coded by the remaining authors (ES and LT). Any discrepancies were discussed until final agreement was reached on theme headings from all authors.

The codes were grouped around the following time points to create overarching themes in the analysis: Pre/early pregnancy (time spanning trying to conceive and the first trimester from conception to 12 weeks gestation); mid pregnancy (second trimester spanning 13–27 weeks gestation); late pregnancy +labour and birth (third trimester spanning 28–40 weeks gestation followed by labour and birth) and postnatal (immediately post-birth up to 12 months). Overarching themes were identified as key timeline points over the perinatal period, and sub themes were created to reflect the views of the perinatal mothers and HCPs along each time point where the information work and perspectives of the perinatal women and HCPs were captured.

Healthcare professional interview transcripts were also thematically analysed and grouped around the perinatal time points to highlight the healthcare perspective of information exchange with perinatal women from early pregnancy to postnatally. During analysis of healthcare professional interviews, it was anticipated that similarities between themes from the perinatal interviews would arise, however data analysis was not constrained by prior themes and author analysis and discussion ensured that the final set of findings reflected the perspectives of HCPs in addition to those of the perinatal women (see Table 3).

**Table 3:** *Timeline themes related to Mothers and HCPs*.

Timeline theme	Information work activities and perspectives	
	Mothers	HCPs
Pre/Early pregnancy This covered the time from trying to conceive up to the end of the first trimester of pregnancy (0–13 weeks gestation).	• Pregnancy self-tracking • Digital information seeking	• Intense information exchange via paper-based tools and resources
Mid pregnancy This reflected the second trimester of pregnancy from 14–27 weeks gestation.	• Information seeking online often followed by in person HCP corroboration	• Tailored information provision. • Digital resource recommendation and signposting
Late pregnancy/ Labour+Birth This related to the third trimester of pregnancy (28–40 weeks gestation) and covered labour and birth.	• Lack of specific information provision • Reliance on online information seeking.	• Reiteration of general information and choices
Postnatal period This related to the period from immediately post-birth to one-year post-birth.	• Shift in information focus towards baby is reflected in the provision of paper-based tools • Online and offline information and support sought. • ‘New mum journal’ valued for reflection and supporting information exchange.	• Transition period important for establishing new relationships for information exchange. • Value paper-based tools and cautiously optimistic about digital replacements.

## 4. Results

### 4.1. Pre pregnancy and Early pregnancy

#### 4.1.1. Perinatal women Perspective

Information seeking supported by technology use begins pre pregnancy. Apps and social media were the main resources used in this period, with women reporting that Pinterest, YouTube and the Ovia app were helpful in adjusting diet and exercise regimes. Self-tracking apps and monitoring personal generated data (PGD) was an important aspect of information work during pre and early pregnancy. One participant reported following healthy ‘fertility’ diets and exercises to ensure the body was ready for pregnancy prior to conception.

“I did loads of searches on fertility, and foods for fertility. I had a Pinterest board and stuff for fertility foods. I had fertility inducing exercises and stuff, so I’d look at fertility yoga on YouTube.” (Amy, 3^rd^ trimester)

Participants also heightened their awareness of their own fertility and engaged with apps to track their menstrual cycles, focusing on ovulation each month to optimise conception.

“I had the OVIA app and that told you when you were ovulating.” (Rebecca, 2^nd^ trimester)

During early pregnancy, before any contact with HCPs, the perinatal women reflected on their experiences of using technology to meet information needs. One new mother spoke of liking the comparisons of baby to fruit on a mobile app and using the app to track bodily changes during the first trimester.

“They are very visual; they are really easy to use. So, the first thing that will pop up is a little picture of what size it is in fruit and then you can name your baby on it. Then it’s just got different articles that pop up every day, so you can read them or not.” (Amy, 3^rd^ Trimester)

“I downloaded the OVIA app, that was the first app I downloaded… I like checking the daily, it’s got a daily thing that pops up about what should be happening to your baby and what you should be feeling at that time and stuff. So, I find that quite helpful.” (Amy, 3^rd^ trimester)

These apps also provided women with information about pregnancy symptoms including mood changes that might occur.

“It’s called Glow. I quite like it actually so it gives you a daily summary of what’s going on, how things are developing and how you might be feeling. That kind of thing.” (Rebecca, 2^nd^ trimester)

Around ten weeks gestation, expectant mothers spoke of having their first appointment with their midwife. One new mother reflected on the first appointment and spoke of not feeling ready to share personal information with the midwife at that stage as a trusting relationship had not yet been built.

“I don’t think they are very sympathetic, and they are quite abrupt in what they are doing. Then they asked me at my first 10 week appointment, they sent my partner out of the room and said (about partner) “are you scared of him, is he emotionally abusive?” and I thought you haven’t built up a good enough rapport with me to ask that question.” (Lauren, 10 months post-birth)

Information is also received by expectant mothers at this stage. HCPs use the first ‘booking appointment’ to provide perinatal women with a lot of information. This usually takes the form of leaflets on topics on diet and exercise, tests and scans, antenatal classes and breastfeeding. This is a wide-ranging set of information, some immediately relevant, some for longer term consultation and is intended to be digested between appointments.

#### 4.2.2. Health Professional perspective

The first appointment with the midwife occurs in the first trimester between 8–10 weeks gestation and is referred to as the ‘booking appointment’. The healthcare professionals discussed how building up a relationship with the client at the antenatal stage was important and provided the client with continuity throughout pregnancy and ideally postnatally. Midwives described the importance of setting up the care structure from the first ‘booking’ appointment and explained how this would set the basis for developing the relationship over the course of the pregnancy. It is hoped that once allocated to a midwife, the clients would receive all their antenatal care from this midwife up until birth.

‘I think it’s really important the first appointment because it’s setting up all of their care and particularly where I worked we tended to caseload the women, so ideally they would be booked by the midwife who was going to look after them for all of their care’- Carol (Midwife)

The booking appointment establishes a space for information exchange. Midwives described using this time to build up a picture of the mother’s personal circumstances, her health and the wider context of social support. Key information elicited by the midwife is recorded in a set of personal paper notes a pregnancy folder that is handed over to the mother to be kept for the duration of the pregnancy. Collecting information from mothers during these appointments helps support ongoing discussions at subsequent appointments.

*‘You get to know them and you get their history, so then going on for further appointments in the future you can then, you know their history and you sort of gain a relationship with them’–Susan (retired midwife).*

### 4.3 Mid pregnancy

#### 4.3.1. Perinatal women perspective

During this period of the perinatal timeline, information seeking, exchange and verification are key activities. In the second trimester (mid-pregnancy) two mothers discussed how the relationship with their midwives starts to develop and they feel more confident sharing personal information. Perinatal women described the importance of continuity of care and developing strong and reliable relationships with their midwives. Being able to see the same HCP at every appointment was an important factor for information exchange, as women relied on their midwife to meet their information needs and regular meetings encouraged the sharing of personal information. One participant, a first-time mother, explained the importance of the midwife relationship:

“Very important because you sort of build up a bit of a relationship with them and you need to feel confident that they know what they are doing. Especially as a first-time mum you haven’t got a clue what’s normal and what’s not so you need feel like whoever you’re seeing knows what they are doing.” (Sam, 4 months post-birth)

“She remembered the baby’s name and was asking about myself and stuff like that, so it’s a bit more personal. But obviously everybody doesn’t get that, but it would be nice if you did, I think, had somebody throughout.” (Amy, 3rd trimester)

Restrictions on time were an impediment to building this relationship and thus had a negative impact on information exchange–especially from the women’s perspectives.

“It almost feels to brief a time you see each other to kind of talk very personally with them.” (Chelsey,1-month post-birth)

During the gaps between HCP appointments, participants undertook intense information work. Women relied upon information from peers online) when the information provided by midwives was not sufficient or if they wanted an answer immediately and did not want to wait for the next appointment as highlighted below in the second extract.

I didn’t find the midwives very informative… I found more on the internet. Mumsnet, I was on Mumsnet constantly, you feel like they answer quite honestly, they go in depth about it, something you’d probably never talk about with the midwife.” (Lauren 10 months postpartum).

“I feel like there are things that I have looked up because I thought ‘oh I don’t know about this’ so it would have been nice to see her a bit more often to check things.” (Isobel, 2^nd^ trimester)

Information from digital resources played a role in midwife appointments during this part of the perinatal timeline. Participants reported sharing information they had found online with their midwife in order to have it verified or simply to have an expert opinion on issues and queries that had occurred between visits and that had prompted women to seek information and answers online.

“I think I checked a couple of things with her, just said I’ve found out this information myself is this true? Because I feel like I need them to clarify it first.” (Alice, 6 months post-birth)

“Yeah, that’s when I asked about the strep B thing, when I’d looked a bit more online about it then I asked her about it. Because otherwise they would never mention it to you.” (Sam, 4 months post-birth)

The focus of bringing this online information into the HCP appointments was primarily to verify information, to seek an expert opinion around issues and queries that had occurred between visits and that had prompted women to seek information and answers online.

#### 4.3.2. Health Professional perspective

HCPs also recognised that clients often sourced information from websites and from apps. It was important from their perspective not to discourage women’s searching for information and taking a proactive interest in their care and that of their baby. Working through information together was important and although it was clear that health professionals were aware of the risks of misinformation, they were careful not to admonish or discourage information seeking but gently pushed a gentle approach is taken to encourage the use of accredited sources that contain evidence-based information to ensure clients are being signposted to the most appropriate sources.

‘I think we would prefer them to use the websites that have all been evidence based and recommended by our trust, and they are not going to be given mixed messages’.- Catherine (Health visitor).

‘of course I’d ask what the site was and what the, if she’d used Facebook and exchanged misinformation you don’t want them to–I’d say oh that’s interesting but the sources I’d use might be XY and Z and state NHS research.’- Catherine (Health visitor).

Specific sources such as the NHS and NICE were recommended among the health professionals and were described as ‘official’ sites where clients would be able to search for accurate and trusted information.

‘If any of the leaflets we gave out they would maybe have a little reference on them, but it would all be cited through NHS and NICE and stuff like that, through official sites you know. Not google’.—Susan (Retired midwife)

The current health visitor also promoted a health professional recommended app to clients. Baby buddy is a pregnancy and parenting app which contains information tailored to both baby and new mothers (and fathers) and is designed by health professionals and accredited by the NHS. The app is designed to give parents additional support and advice in the first five years of their child’s life.

‘baby buddy, that was an app that (North East) NHS trust invested a lot of money in to, and we still give people the information about baby buddy at the moment, and there’s lots of links into other searches and things like that which are all evidence based as well’- Catherine (Health visitor).

### 4.4 Late pregnancy/ Labour & Birth

#### 4.4.1. Perinatal women Perspective

Towards the end of pregnancy, participants reported feeling they lacked detailed information about labour and birth and what to expect in the first few weeks after the baby arrives. Information provided at NHS-based antenatal classes did not help to prepare expectant mothers after the baby arrived:

“But again, it was very skimmed over, we did a breastfeeding class and I came away and said to my mam ‘I still don’t know what to do, until I have the baby I don’t feel like I’m going to know what to do’. And I didn’t, I didn’t have a clue, when I tried breastfeeding afterwards, I thought ‘this is nothing like what they explained in the class.” (Lauren, 10 months post-birth)

Women expressed concern over the timing of the information and the level of detail provided. Much of this information was provided in group sessions rather than one-to-one. Despite midwife appointments becoming more frequent in the later stages of pregnancy, our perinatal women felt that this period of the timeline was relatively sparse in terms of information provision. Appointments with midwives focused on medical data with checks on baby and mother taking precedence over and information provision. At this point, women often felt they had to engage with digital resources to seek further information. This helped expectant mothers feel more empowered and knowledgeable about what to expect during labour and birth.

“I think looking on the internet that was what prepared me for what was about to happen. I got started off, even though I didn’t know I was getting started off I had read everything about getting started off so when they were coming in and saying, ‘we are going to do this now’ I thought oh I already know that, that’s fine.” (Lauren, 10 months post-birth)

“So, my preparation for labour came from stuff I’ve found myself, and people that I’ve spoken to. I’ve got a book on hypnobirthing and once I got pregnant and I had lots of people messaging saying ‘have you looked into hypnobirthing? Someone I knew did it.’, so I looked into all that myself.” (Claire, third trimester)

#### 4.4.2. Health Professional perspective

The retired midwife spoke of information provision to women relating to birth and expectant mothers usually having a choice of where to have their baby. They would be provided with information about specific units or the possibility of home births. The retired midwife also explained that expectant mothers would have an option to visit their chosen hospital unit however this was not reiterated from the current practising midwives.

‘They got a choice of what hospital they wanted to have the baby and whether it was a home delivery, or hospital and they got what hospital they were hoping to choose…. broad spectrum of information leaflets and we went through basically what her care would be and how we would be available for them for the next, til the delivery…. things like testing and the hospitals they would look etc. at the time we could visit the hospitals but mostly did that once they were nearer delivery’- Susan (retired midwife).

### 4.5 Postnatal

#### 4.5.1. Perinatal women Perspective

During the postnatal period there is a transition in HCP care from midwife to health visitor and this can provide challenges in terms of information work from both perspectives. Both mothers and HCPs felt that ideally continuity of care should extend into the postnatal phase, this way new mothers would be familiar with their healthcare team. This transition in care was potentially difficult for new mothers and the importance of developing a new and strong relationship with the health visitor was emphasised.

“Then obviously after you’ve had your baby your sort of very vulnerable and you know, there’s lots of changes going on. So you need a nice health visitor so again you feel you can talk to, or is supporting you in whatever you’re doing and keeping you right. Because it’s quite a scary time really.” (Sam, 4 months post-birth)

While these relationships allowed a safe environment for information exchange across a diverse range of topics, many women felt that the focus of the information exchange had now shifted to their baby, and this left some new mothers feeling left out and with unmet information needs.

‘I don’t think I got anything on caring for my stitches or caring for the bleeding or even like signs to look out for baby blues, and what to do but nothing so you just sit and look on the internet for it all.’- (Rachel -from follow up study)

Under these circumstances, new mothers tended to turn to digital sources to meet their information needs. Information about postnatal recovery, the physical and mental health aspects, was the key need and new mothers sought this information online.

‘One day because I was feeling low and kept crying and I thought oh god have I got postnatal depression, so I googled it’ (Rachel- from follow up study)

Once the baby has arrived, however, the range of information sources increases with family and friends becoming more involved in seeking and sharing information and this builds on the role of the health visitor.

‘The health visitor has been really useful, she has given loads of good information. And she’s been really real with stuff rather than saying like jazzing it up a bit, she has been really honest with stuff. But yeah, I think mainly friends you can feel a bit more relaxed asking silly questions.’- Rachel- (from follow up study)

The pregnancy notes folder is collected back by the HCP soon after birth. The sense of loss experienced by new mothers when their pregnancy notes folder was taken away during the last midwife visit was noted by participants and signalled the end of that period of the timeline.

“I was like ‘oh do I not get to keep it?’ because as much as I didn’t understand what was in it, it would have been nice to look back on it. I think especially second time it would have been nice to compare the appointments and stuff with them. But no, I didn’t realise they took it off you, and I’m quite sentimental I like to keep stuff.” (Nicola, 12 months post-birth)

“The midwife on the last appointment was like ‘right I’ll just take this’, she was like ‘I’ll give you all the loose stuff in case there’s anything you need’ and then she just took it and you were like… oh. And Mike was like I feel quite sad because that’s been with us for the whole journey, and she just took it.” (Lauren 10 months postpartum)

In our follow up interviews, the participants valued the ‘new mum journal’ we gave them to record information, questions, data and experiences about baby and themselves. Both participants enjoyed having a dedicated place to record their thoughts and feelings themselves and baby for future reference. The journal also became a way to maintain focus on both mother and baby and having this information stored together allowed mothers to make connections between their own experiences and what was happening with their new babies. Looking back at the journal allowed mothers to document and reflect on their progress in dealing with the challenges of motherhood.

‘I like that it is about Rosie and that it’s also about me. Like I said I feel like I’ve forgotten how I felt at the beginning to how I feel now like how sore I was and even like emotional… even looking back at that to think actually I’ve come so far from that first week.’- (Claire- from follow up study).

I think I had a few things about wind and her tummy, and I think I had recorded things in so then I’d be like she’s had bad wind for, oh right I’ll have a look and this is where she starting having issues or whatever. Like stuff like that, so I could relate back to things and look at the specific weeks’- Rachel (from follow up study).

Digital versions of the ‘red book’ (the child’s health record book) and the ‘new mum journal’ were discussed with our follow up participants. While the paper-based journal had initially been a welcome distraction from smartphone apps, both parents conceded that an app or online journal would be more practical especially as baby got older. Issues of trust and security were also important to both mothers when discussing any digital form of information exchange.

‘I think it would be a trust issue of, is the technology going to keep my information safe? I think that would be the only issue that I would have. Especially if you were putting photos onto an app…So yeah that would be my only thing I’d have to make sure it was really safe to store information.’- (Claire- from follow up study)

#### 4.5.2. Health Professional perspective

When the midwife hands over care to the health visitor at 10–14 days postnatal, there is little verbal communication between the health professionals, information about the mother and baby is exchanged as written communication. A ‘discharge sheet’ or ‘form’ is filled out by the midwife and stored in the red book to be picked up by the health visitor at their primary home visit.

‘It may be that we don’t see the midwife at all, they would complete in the personal child health record book that red book, they would complete their transfer sheet and they also do a discharge sheet for health visitor’.- Catherine (Health visitor).

Information stored in the red book covers areas from children’s health development, e.g., record of immunisations, to development such as first tooth and smile. It can be a useful source to both record and store all of baby’s early development milestones and be used by the health professionals to keep track of their health and medical progress.

‘So, the red book we try to encourage people to really look at it as a great resource, a record of all their baby’s kind of checks and immunisations, their growth and something that benefits them as well as health professionals. There is obviously the developmental sort of drawings in the back, first smile and tooth and things like that and there’s lots of really good information in there as well. So yeah, obviously we use the red book to record any of the routine reviews that we have as well.’–Catherine (Health visitor).

One health visitor explained that the red book is a two-way information source and can be used to facilitate communication and shared understandings. The red book provides a focus for the information work of the HCP appointment.

‘For me I think a lot of people were really anxious when that health record came out, but for me it was the basis of working together with the family. I try at the end of the meetings day well how shall we record this visit, so that I’m writing something down that somebody it’s usually the mother, that the mother could sign up to, yes that is I think what I think we set out to do and it’s what we’ve achieved.’ -Kate (Retired health visitor).

In terms of digital tools, HCPs believed a digitised ‘new mum journal’ or similar tool could be beneficial for new mothers and HCPs. A digital tool could encourage new mothers to ask questions ahead of meetings and for HCPs to have a better sense of mothers’ concerns and questions in advance of meetings. When asked about a ‘new mum journal’ as a tool to encourage information exchange, one current midwife replied:

“Yeah it probably would be good for them to have a journal so you could read up on what they had been struggling on, it helps them to remember if they had any questions because it’s very on the spot when you go out and it’s like I feel like I had so many questions to ask you but I’ve forgotten them because you are suddenly put under pressure.’–Lindsey (Midwife).

‘I think it would be quite helpful for things like that to have a journal so you could look at it and say oh right so you said you were struggling with this have you got any questions and can I help you in anyway with it?’ –Lindsey (Midwife).

The HCPs did however discuss concerns around digital exclusion as well as data security issues with any new digital resources.

‘ I would worry about a perspective of people who don’t have acess to the internet and dont have a phone, couldn’t access things online, I think that would be more my worry in terms of just incase if someone didn’t have access to that information and then it would be nicer for them to have a handheld *copy’- Lindsey (Midwife).*

‘If you have got an electronic version there’s less chance of things going astray and being sort of oh well now we haven’t got all the information that we need. I think certainly there could be some challenges around who has ownership of that information electronically.’- Carol (Midwife)

## 5 Discussion

This paper has explored information work that perinatal women undertake from before they discover they are pregnant to six months after the birth of their baby. Our findings show that information work during this period consists of many information related activities including seeking, monitoring, recording, questioning, sharing, and checking and that it varies considerably over the perinatal period, shifting back and forth in focus between mother and baby. We note the importance of HCPs as stakeholders in information work and finally we note that while digital support for information is useful, paper-based resources are still an important shared resource allowing reflection and supporting communication. We discuss our findings in the light of existing research before suggesting design considerations for digital tools to support perinatal women.

Our findings show the changing nature of information work over the perinatal period and the role and importance of digital and paper-based resources for supporting this work. During pre-pregnancy and early pregnancy, information work centres on exploring digital resources for information seeking and for generating personal generated data (PGD) that can be noted and tracked. Information work here is self-contained, does not require sharing and is driven and supported by the digital technology. During mid and late pregnancy, information work takes place against a backdrop of structured appointments in which trusted relationships facilitate information exchange. During these intense periods of activity, information is shared between perinatal women and HCPs and PGD and other information is noted verbally and recorded in paper-based tools. Information is shared with women via paper-based resources. In keeping with previous research [6] we found that digital resources are used to supplement information provided during early prenatal visits with HCPs. However, we found that between these appointments information work is more complex and involves seeking and evaluating information from HCPs and online sources but also reflecting on experiential information, monitoring, and tracking personal data about, inter alia mood and sleep. Long gaps between appointments fuel ‘in the moment’ access to information and support [28]. During the postnatal period information needs multiply with perinatal women having questions and concerns relating to themselves and to their new baby. Overall, postnatal appeared to be the most vulnerable stage in the motherhood journey and this confirms previous research that has identified a heavier focus on the health and wellbeing of the baby during early postnatal contact with HCPs[8] and the state of the ‘invisible mother’ [22] where many postnatal mothers feel less supported by HCPs. The transitions around HCPs, the loss of pregnancy notes and a clear differentiation between ‘mother’ and ‘baby’ needs highlighted gaps in support for information seeking and exchange.

### 5.1 Digital support and paper-based tools for perinatal women

A perhaps unexcepted finding was that paper based resources were commonly used and valued as part of the information work of pregnancy and new motherhood. The pregnancy notes as a curated source of information were seen as something of importance to mothers. The information in the notes combines information which has been offered by mothers as well as information provided by and ‘revealed’ by HCPs through test results and baby monitoring. It documented and represented their pregnancy and there was a certain sense of ownership over the information. This builds on the autoethnographic work of Papen [29] who documents her changing relationship with her pregnancy notes. At the start of her pregnancy, she sees herself as an administrator rather than owner of the notes yet by the end of her pregnancy recognises them as a physical symbol of her new identity. As new mothers transition into their role as caregiver there appear to be fewer opportunities for ‘mother centred’ information exchange and the loss of the pregnancy folder accentuated this point. The pregnancy folder had provided a resource for information exchange and the sharing of personal data between the HCP and our participants. While new mothers receive a ‘red book’ to document weight gain and record immunisations, there is no equivalent for mother centred information and thus a clear divide between information curation around ‘mum’ and ‘baby’ is established soon after baby arrives. So, at different time points the focus of the information work was clearly on either mother or baby.

The role of digital technology to support information work was woven through the timeline. From pre pregnancy to information seeking online post birth, digital resources were important. Digital resources were sometimes used instead of (or before) HCP support. Mobile apps in particular focused on monitoring and tracking ovulation and baby development although few such apps are of high quality and often limited in the scope of information they provide [14]. Confirming earlier research [23], our participants explained their main preparation for labour came from online sources and although NHS antenatal classes were provided, the information was deemed basic, and mothers felt this had not prepared them for the realities of labour and postnatal recovery. A lack of postnatal education poses many risks for women in the postnatal period [30].

For perinatal women, information work also involves the integration of different sources and the corroboration of information. Women checked information sought online with HCPs and followed up midwife and health visitor appointments with online information seeking. The process of keeping information in mind ready for the next appointment and cross checking the information can both be seen as forms of information work. This finding emphasises the importance of integration of digital sources with in-person support across a range of health contexts and adds to earlier research suggesting that multiple, integrated forms of support are important in health decision-making [31].

Finally, we extend previous work by highlighting the importance of self-reflection on information as a form of self-care. We suggest that this forms an important part of information work. Using information recorded to curate experiences and reflect on patterns of change or stability [32]. The new mum journal allowed both reflection and encouraged communication and the journal prompted support and information seeking directly from HCPs which has potential to improve information exchange. The journal acted as an unseen mediator of communication in the sense that mothers viewed the information stored in the journal prior to appointments with HCPs and used it to prompt questions and to perform a sense check around their experiences before engaging with HCPs. This encouraged new mothers to seek support direct from HCPs, a process which not all mothers find straightforward [24].

### 5.2 Considerations for digital tools to support perinatal women

Exploring information work during the perinatal period from different perspectives provides a sharper focus on the pinch points for information exchange as well as potential opportunities to support information work across pregnancy and early motherhood. Our findings suggest several design considerations for digital tools to support perinatal women. We have split these into considerations for pregnancy and for the post-natal period and they are outlined below.

#### 5.2.1. Pregnancy

Design considerations here should centre on features to support the development of trusting relationships between perinatal women and HCPs. More frequent, lightweight touchpoints supporting ease of communication and contact. Perinatal women valued the information provided usually in paper form by HCPs at booking appointments, but it sometimes felt overwhelming and not always time critical. Information about different hospitals, about labour and birth for example could be delivered via a digital tool at more timely points along the perinatal journey. Signposting to suitable, credible digital resources could be achieved via the digital tool to model reliable sources of digital information. Ideally any new digital tool would have messaging capability that would facilitate interactions between perinatal women and HCPs between appointments. This functionality would allow simple queries to be resolved quickly. In the current context of stretched resources, providing additional time to respond to digital queries may be difficult although potentially could free up time from additional phone consultations. It may be more feasible to consider a tool that supports information checking and corroboration. Women had to remember information taken from online sources until it could be checked with the midwife or note down questions before appointments. A memory aide tool that helped women capture online information and its source as well as being able to annotate the information with comments and questions could support information exchange during appointments. HCPs would be able to see the source of the information and use the opportunity to signpost to alternative sources as appropriate.

#### 5.2.2. Postnatal period

The focus of information work especially during the postnatal period is heavily integrated with issues for baby and for the mother being concomitant. Design considerations should focus on crafting digital resources to support mother and baby together. For example, a digital space that supports information exchange and reflection around a mother’s mental and physical health could benefit from contextual information around baby’s sleep and development. The new mum journal explicitly provided space for recording and reflecting upon information about mother and baby. It also provided a space to capture personal generated data and information that had been sought out online or from other sources such as friends and family. The journal allowed mothers to write about and reflect on their own journeys under the more familiar guise of documenting baby’s progress. The journal felt easy to review partly because of the paper format but our participants suggested there would be practical benefits of a digital version in terms of ease of use and familiarity with digital tools.

Creating a safe space for new mothers to upload health information about their baby could allow the curation of information which prompts future discussions with HCPs. Opportunities to capture information about mother and baby together are valued. Digital baby books or e-red books in the UK have previously been trialled in some locations [33] and it will be important to understand more about how issues of trust, privacy and security affect their adoption and continued use. Future systems could encourage mothers to store and save information about themselves as well as their baby to document this unique experience. Being able to curate information, reminisce and reflect on that information may assist mothers in seeking support when needed as this information could be shared with health professionals during post birth home visits. HCPs may have time to review these digital resources before appointments to identify areas for further support for new mothers to promote positive postnatal mental health and help them to feel less vulnerable after giving birth. If it is not possible for HCPs to view the resources in advance, going over the resources together at appointments might provide a series of useful prompts for discussion. Our findings add to this literature by corroborating that infrequent antenatal appointments can inadvertently create opportunities for good information exchange.

### 5.3 Overall design considerations

Our perinatal women participants often referred to their information seeking needs rather than their sharing needs when discussing technology and it may be that as women are pushed towards technology resources that important opportunities for sharing may be missed. Sharing PGD may feel less appropriate outside of the trusted HCP relationship and raises very real concerns around trust security and privacy, indeed both our perinatal women and HCPs expressed some concerns over data storage issues in relation to new digital tools. In reflecting on how we want technology to shape this future space we need to consider how women may want to seek control of their privacy settings choosing when and with whom to share information about themselves and their baby (see for example [34] examining privacy controls around the sharing of other sensitive data We also need to reflect further on the issue of psychological ownership in relation to information, especially PGD and think about access issues in respect to data held digitally [35]. Finally, issues around digital literacy and inclusivity around disability and language need to be considered going forward and are often overlooked aspects of new digital tools [36]. This is an important potential barrier to implementation for both perinatal women and HCPs. The co-design of new tools with relevant stakeholders will be key to their uptake and use. Paper based tools that are flexible and allow for different ways of capturing and presenting information may retain value for many people [37,32].

### 5.4 Limitations

The perinatal women in our study were predominantly based in the in Northeast of England and our study reflects how information and support is provided to women in this region of the UK. All our participants in this sample were White British and in a relationship, and as such it would be useful to know how perinatal women from various cultural and socioeconomic backgrounds experience information and support in this region, and how single mothers navigate information work in pregnancy and motherhood. Due to the sample size and the fact that some participants were approached to take part in the study through the author’s personal networks, there is potential for selection bias to occur, and this limits the generalisability of the current findings. Future research would benefit from including a representative sample to capture perinatal women and HCPs from different cultural and socioeconomic backgrounds across the region.

It was apparent from the follow-up interviews that both first time mothers would have liked to continue with the journal beyond the three-month period of the study. This suggests that there may be value in a longer study examining information work over the first year of motherhood especially for first time mothers. Future work also needs to examine the value of digital tools for second time mothers.

A strength of this study was the incorporation of a health professional perspective. Expanding this perspective to include other health professionals involved with antenatal and postnatal care for example, general practitioners (GPs), breastfeeding support workers or specialist nurses would provide additional insights into the role of HCP stakeholders in the information work of perinatal women.

Although we recognise that the UK context for this work may differ from other locations in terms of the precise pattern of HCP interactions, the information needs, and technology use findings of the study are likely to be growing issues across a diverse range of settings as cost and time pressures for HCPs increase. Digital resources look set to become an increasing part of the information work of perinatal women.

### 5.5 Future research

Future research should focus on examining the perspectives of a larger sample of participants including HCPs from various regions in the UK to compare how information and support provided in the Northeast may differ to other areas based on local practices and resources. Design workshops focusing on the considerations outlined above involving a range of different stakeholders and importantly perinatal women from a range of different backgrounds would keep the focus on inclusivity going forward. As the sample of perinatal women were mainly first-time mothers, future work could expand the findings to include a larger sample of second-time mothers to further understand how they are using digital information tools to seek support and information. This could highlight where current information gaps are and provide a focus for HCPs to tailor support and information provision to perinatal women. Future work would benefit from exploration of digital health literacy for perinatal women and HCPs from across varying socioeconomic backgrounds to ensure that access to and knowledge of digital information tools could be accessible to all new mothers.

## 6. Conclusion

Information work for women varies across the perinatal timeline. Challenges exist at key transition points and reflecting on more integrated digital resources that support information work focused on mother and baby may also have a role to play in enhancing communication between perinatal women and healthcare professionals.

## Supporting information

S1 Text Example follow up interview text. Midwife interview schedule. Perinatal women interview schedule. Health visitor interview schedule.
(PDF)

## References

[1] Lee Y, Moon M. Utilization and content evaluation of mobile applications for pregnancy, birth, and child care. Healthcare informatics research. 2016 Apr 30;22(2):73–80. doi: 10.4258/hir.2016.22.2.73 27200216 PMC4871848

[2] Demirci JR, Cohen SM, Parker M, Holmes A, Bogen DL. Access, use, and preferences for technology-based perinatal and breastfeeding support among childbearing women. The Journal of perinatal education. 2016;25(1):29. doi: 10.1891/1058-1243.25.1.29 26848248 PMC4719111

[3] Lupton D. ‘It just gives me a bit of peace of mind’: Australian women’s use of digital media for pregnancy and early motherhood. Societies. 2017 Sep 15;7(3):25.

[4] Johnson SA. “Maternal devices”, social media and the self-management of pregnancy, mothering and child health. Societies. 2014 Jun 13;4(2):330–50.

[5] Javanmardi M, Noroozi M, Mostafavi F, Ashrafi-Rizi H. Internet usage among pregnant women for seeking health information: a review article. Iranian journal of nursing and midwifery research. 2018 Mar 1;23(2):79–86. doi: 10.4103/ijnmr.IJNMR_82_17 29628953 PMC5881235

[6] Lupton D, Pedersen S. An Australian survey of women’s use of pregnancy and parenting apps. Women and birth. 2016 Aug 1;29(4):368–75. doi: 10.1016/j.wombi.2016.01.008 26874938

[7] Larsson M. A descriptive study of the use of the Internet by women seeking pregnancy-related information. Midwifery. 2009 Feb 1;25(1):14–20. doi: 10.1016/j.midw.2007.01.010 17408822

[8] McLeish J, Harvey M, Redshaw M, Alderdice F. “Reassurance that you’re doing okay, or guidance if you’re not”: a qualitative descriptive study of pregnant first time mothers’ expectations and information needs about postnatal care in England. Midwifery. 2020 Oct 1;89:102813. doi: 10.1016/j.midw.2020.102813 32798075 PMC7493710

[9] Alderdice F, McLeish J, Henderson J, Malouf R, Harvey M, Redshaw M. Women’s ideal and real expectations of postnatal care during their first pregnancy: An online survey in England. Midwifery. 2020 Oct 1;89:102815. doi: 10.1016/j.midw.2020.102815 32829965

[10] NICE.Postnatal care. NICE; 2021. Available from: https://www.nice.org.uk/guidance/ng194

[11] Corbin J, Strauss A. Managing chronic illness at home: three lines of work. Qualitative sociology. 1985 Sep;8(3):224–47.

[12] Dalmer NK, Huvila I. Conceptualizing information work for health contexts in Library and Information Science. Journal of Documentation. 2020 Jan 7;76(1):96–108.

[13] Mazanderani F, Hughes N, Hardy C, Sillence E, Powell J. Health information work and the enactment of care in couples and families affected by Multiple Sclerosis. Sociology of health & illness. 2019 Feb;41(2):395–410. doi: 10.1111/1467-9566.12842 30677163

[14] Brown HM, Bucher T, Collins CE, Rollo ME. A review of pregnancy apps freely available in the Google Play Store. Health Promotion Journal of Australia. 2020 Sep;31(3):340–2. doi: 10.1002/hpja.270 31225924

[15] Grimes HA, Forster DA, Newton MS. Sources of information used by women during pregnancy to meet their information needs. Midwifery. 2014 Jan 1;30(1):e26–33.Henshaw doi: 10.1016/j.midw.2013.10.007 24246969

[16] NHS. Midwife [Internet]. Health Careers. NHS; 2015. Available from: https://www.healthcareers.nhs.uk/explore-roles/midwifery/roles-midwifery/midwife

[17] Weston C, Anderson JL. Internet use in pregnancy. British Journal of Midwifery. 2014 Jul 2;22(7):488–93.

[18] Newhouse N. bump2bump: Online Peer Support in First-Time Pregnancy. InProceedings of the 2016 CHI Conference Extended Abstracts on Human Factors in Computing Systems 2016 May 7 (pp. 239–243).

[19] Lebert-Charron A, Wendland J, Vivier-Prioul S, Boujut E, Dorard G. Does perceived partner support have an impact on mothers’ mental health and parental burnout?. Marriage & Family Review. 2022 May 19;58(4):362–82.

[20] Antoniou E, Stamoulou P, Tzanoulinou MD, Orovou E. Perinatal mental health; the role and the effect of the partner: a systematic review. In Healthcare 2021 Nov 18 (Vol. 9, No. 11, p. 1572). MDPI. 10.3390/healthcare9111572 PMC8624285 34828618

[21] Price SL, Aston M, Monaghan J, Sim M, Tomblin Murphy G, Etowa J, Pickles M, Hunter A, Little V. Maternal knowing and social networks: understanding first-time mothers’ search for information and support through online and offline social networks. Qualitative health research. 2018 Aug;28(10):1552–63. doi: 10.1177/1049732317748314 29281945

[22] de Vries B, LeBlanc AJ, Frost DM, Alston-Stepnitz E, Stephenson R, Woodyatt CR. The relationship timeline: A method for the study of shared lived experiences in relational contexts. Advances in Life Course Research. 2017 Jun 1;32:55–64. doi: 10.1016/j.alcr.2016.07.002 28584522 PMC5454772

[23] Henshaw EJ, Cooper MA, Jaramillo M, Lamp JM, Jones AL, Wood TL. “Trying to figure out if you’re doing things right, and where to get the info”: parents recall information and support needed during the first 6 weeks postpartum. Maternal and child health journal. 2018 Nov;22:1668–75. 29978309 10.1007/s10995-018-2565-3

[24] Ghiasi A. Health information needs, sources of information, and barriers to accessing health information among pregnant women: a systematic review of research. The journal of maternal-fetal & neonatal medicine. 2021 Apr 18;34(8):1320–30. doi: 10.1080/14767058.2019.1634685 31216921

[25] Sayakhot P, Carolan-Olah M. Internet use by pregnant women seeking pregnancy-related information: a systematic review. BMC pregnancy and childbirth. 2016 Dec;16:1–0. 27021727 10.1186/s12884-016-0856-5 PMC4810511

[26] Dobele A, Fry J, Rundle-Thiele S, Fry T. Caring for baby: what sources of information do mothers use and trust?. Journal of Services Marketing. 2017 Nov 22;31(7):677–89.

[27] Braun V, Clarke V. Using thematic analysis in psychology. Qualitative research in psychology. 2006 Jan 1;3(2):77–101.

[28] Facca D, Hall J, Hiebert B, Donelle L. Understanding the Tensions of “Good Motherhood” Through Women’s Digital Technology Use: Descriptive Qualitative Study. JMIR Pediatrics and Parenting. 2023 Oct 25;6(1):e48934. doi: 10.2196/48934 37878372 PMC10632912

[29] Papen U. Pregnancy starts with a literacy event: pregnancy and antenatal care as textually mediated experiences. Ethnography. 2008 Sep;9(3):377–402.

[30] Walker SB, Rossi DM, Sander TM. Women’s successful transition to motherhood during the early postnatal period: A qualitative systematic review of postnatal and midwifery home care literature. Midwifery. 2019 Dec 1;79:102552. doi: 10.1016/j.midw.2019.102552 31605940

[31] Bussey LG, Sillence E. The role of internet resources in health decision-making: a qualitative study. Digital Health. 2019 Nov;5:2055207619888073. doi: 10.1177/2055207619888073 31741741 PMC6843735

[32] Claisse C, Kasadha B, Stumpf S, Durrant AC. Investigating daily practices of self-care to inform the design of supportive health technologies for living and ageing well with HIV. In Proceedings of the 2022 CHI Conference on Human Factors in Computing Systems 2022 Apr 29 (pp. 1–19).

[33] O’Connor S, Devlin AM, McGee-Lennon M, Bouamrane MM, O’Donnell CA, Mair FS. Factors affecting participation in the eRedBook: a personal child health record. In Nursing Informatics 2016 2016 (pp. 971–972). IOS Press. 27332437

[34] Bussone A, Stumpf S, Bird J. Disclose-it-yourself: security and privacy for people living with HIV. InCHI EA’16: Proceedings of the 2016 ACM annual conference on Human Factors in Computing Systems Extended Abstracts 2016 May (pp. 1–4).

[35] Morewedge CK, Monga A, Palmatier RW, Shu SB, Small DA. Evolution of consumption: A psychological ownership framework. Journal of Marketing. 2021 Jan;85(1):196–218.

[36] Lau Y, Wong SH, Cheng LJ, Lau ST. Exploring experiences and needs of perinatal women in digital healthcare: A meta-ethnography of qualitative evidence. International Journal of Medical Informatics. 2023 Jan 1;169:104929. doi: 10.1016/j.ijmedinf.2022.104929 36435014

[37] Ayobi A, Sonne T, Marshall P, Cox AL. Flexible and mindful self-tracking: Design implications from paper bullet journals. In Proceedings of the 2018 CHI conference on human factors in computing systems 2018 Apr 19 (pp. 1–14).

