# Supplementary material for: Information work and digital support during the perinatal period: Perspectives of mothers and healthcare professionals
Source: PLOS Digit Health. 2024 Aug 16;3(8):e0000387. doi: 10.1371/journal.pdig.0000387 (PMC11329105; doi:10.1371/journal.pdig.0000387)
Supplement: S1 Text — Midwife interview schedule. Perinatal women interview schedule. Health visitor interview schedule. (PDF) [file pdig.0000387.s001.pdf]

Supporting information

1. Example transcript from follow-up interview

Case Study- 1 month interview (Participant 1)

Key:

R: Researcher

P: Participant

R: How have you found using the new mum journal?

P: I've enjoyed it. It's like.. I use that and a one that a friend got me, but that one's a daily one and this one's a weekly one. But it's.. obviously with the weekly one it's easier because with the daily one I don't fill it in every day, I look at it and go 'oh god I haven't done that for a few days' or whatever. So the weekly one it's really easy to use and it's quite nice to reflect on your week to see what you've done, what your feelings are, er.. but I find it quite cathartic. Is that the word?

R: Yes it is, I like that word. Is there anything you would have wanted to write in the journal but there hasn't been space for it?

P: Erm.. let's have a look a the page… erm I don't think so. So things I would want to write but I haven't.. maybe there could be like.. I suppose that's highs and lows though like a 'how baby is feeling this week' or something. But no I wouldn't say there's necessarily anything I want to write down because it covers everything. The bit I tend to struggle with is the 'things to do' bit.

R: Yeah, well that's fine because I suppose every week your list of things to do will be different. So how do you feel having a diary where you can store personal information about yourself rather than it just being about baby?

P: I like it. So how do I feel?

R: Yeah so do you like having this to store your own information in or..?

P: Yeah I think it's quite useful to have baby and mam stuff together. So say if it was all.. like it comes hand in hand I suppose. Erm so like, the highs and lows, yeah I suppose it tends to be about us both but it is useful having stuff for me aswell because that's quite therapeutic I think. Because sometimes when you write things down you don't realise how you are feeling about something until you write it down then you are like 'oh right ok' then that can make it better for baby stuff. Things like that, definitely for the highs and lows because sometimes I've sat and had to go through my week and I've though 'oo I don't actually know what my low point is or what my high point is. Then the how mam's.. actually that's a good one the 'how mam is feeling' erm, because you sort of don't know, you get to a point where you think oh how have I felt this week?

R: Yes because you are more focused on baby.

P: Yeah, and it's good to sum it up in a week, because saying if you were doing a day-to-day thing, like you'd focus on, I don't know you'd know how you feel in a day but if you get through a whole week you can kind of sum it up. So say if you've had a few bad days but then you go right well overall this

week.. you tend to go oh actually it’s been a nice week. So it’s not focusing too much on the down points if you were doing it every day.

R: Oh that’s good. Have you showed this to anyone, or would you?

P: Only you. Yeah actually.. did my mam have a little look?.. I probably would. I don’t know if I would show my other personal one to other people because that feels more like a ‘dear diary’ sort of things, that’s like me just scribbling away thoughts. But this because I know that you are going to see it or it could be seen then it’s more like.. not less personal because I still feel like I share it but it’s more structured and it would be like.. it’s me writing stuff in that I would be fine for other people to see.

R: Right ok. So are you most likely to share information about yourself with others? So with health professionals or family and friends, are you more likely to talk about how you are feeling, or how your experiences of motherhood are, or are you more focused on talking about baby?

P: Probably baby, but I think the first couple of weeks, definitely the first week I felt it was me, because I felt she was quite easy all she did was sleep and feed so I thought oh brilliant. Then a lot of it was about me so I felt like a lot of the medical advice I sought from the midwife and stuff was about my recovery and what I need to do, or like mental state and things like that and that probably trickled into the second week. But now that I’m feeling better it’s not talking about her to people.

R: So thinking about all types of information, so I know you said you have another diary, what have you found most useful relating to yourself? That could include information you’ve been given or information you’ve found online.

P: Talking to friends, erm,. probably just google for general things. There’s not anything specific that I’ve used but mainly the internet if I’m looking for stuff. In terms of recovery stuff, I text the student midwife loads, so that’s who I would speak to, so when it was like about my bleeding or if it was anything about, like even changing the bottle teats up. Because she’s available and she’s a professional and I’ve got her number, even though we’ve been discharged I still text her about stuff. I even did message her about breastfeeding stuff, so I’d say so was the main thing when I thought ‘oh I’ll just check that with someone’.

R: That’s really good. What would you say the benefits or what have you enjoyed about this (journal) being a paper copy?

P: I think because properly writing stuff, because you’ll be on your phone all the time so that doesn’t feel like.. I think id do it in less detail if it was on my phone and you wouldn’t sit and spend time doing it. So normally if I know if I know I’ve got to do that it will be a nice ten minutes where I think I’ll grab a cup of coffee and I’ll sit and write in that and my other one as well. Whereas if it was just on my phone, I’d probably forget or I would probably feed her then probably do something and I’d probably do it in bits and not take notice so that’s like a moment to pause.

R: So it’s the actual writing process?

P: Yeah the actual writing of it. I think because you don’t write a lot, but I quite like to physically write things like in journals and stuff because it’s like.. I don’t know it’s like a mindfulness thing isn’t it. So I like to write in and then I like to have it to look back through it, rather than if you had it on an app or something I don’t think I’d properly look back through it.

R: Oh right ok. So my next question was going to be would you prefer or use a digital version of this? Or what do you think a digital version could give you that this doesn’t give you or vice versa?

P: Yeah. So maybe like.. I like sitting writing but maybe people would prefer a digital version because it's easier and you don't lose it. But I make sure I keep that in my changing bag, so then I'm like right I know it's in there. But I suppose you could go back an edit it if you had a digital version and I suppose you could add things as you went along. So I'll do it at the end of the week and try and reflect on my week but if you had a digital version you could just add something quickly. So there's pros and cons, you could get more information into a digital version maybe because there might be more space, then you've always got it and you're not going to lose it.. but I like to still write.

R: Is there things you would include if it was a digital version that you cant include in this? Links to other websites or photos?

P: Yeah maybe there could be, because you know here there's space for photos, I don't print out photos so I wouldn't put any in there. I've even got baby record books where I haven't printed out any photos to stick in yet. But there they could have a space which said 'photos from this week' then you could upload all of your photos from that week which would be nice, and the stuff that says what we did then you could upload the photos of what you did.

R: Yes that would be really nice.

P: Then 'things to do' it could have, I don't know say, you know how there's little milestones every week for babies, there could be little reminders that pop up say like 'have you registered the birth' or 'have you booked your baby in for the 6 week check'. It could be general things that help you remember and think oh no I haven't don't that, or 'have you signed up to a local group' it could be things like that for 'things to do'.

R: Yes that would be good. Would you use social media to share any of this information? Information about yourself.

P: Yeah because I don't mind so much, I would divulge properly and be like 'this week I was really low and feeling like this' but if I felt like it was going to help somebody or like erm, yeah if there was something I felt really strongly about or if I was feeling a certain way or if other people would be interested then I would maybe share something from it.

R: Do you feel like the stuff you do post of social media is more about baby?

P; Yeah at the minute it's just photos of her, it wouldn't be anything to do with her journey or anything, just photos of her. Or maybe like journey in terms of 'this is where she's been today or this is what outfit she's got on' it would be like ' here's Iris and today she tried a new bottle, or today she came off breastfeeding'.

R: Do you feel you wouldn't share that because it's more personal, or privacy issues, would you trust putting that information online?

P: I probably just wouldn't want other people to know what was going on so you just put on little pictures, but again its like a privacy thing.. I suppose you put the celebratory things on social media not like the bad stuff.

R: So my next question.. is the information you keep in the journal separate from what you share online?

P: Yes. There may be a random thing that I'd share like 'what we did this week' or Iris being born or like maybe a few little bits, but yeah I wouldn't share a lot of it.

R: Right. (looking at journal) so you've used a white noise app and the Wonder Weeks app, how have you found those?

P: The wonder weeks is really good I've only had that for a week or two. That you just use every week because it tells you what's happening during that week so that's really good. She's going through a leap at this moment so everything it says about like she might be a bit more clingy, or her digestive system is sorting itself out, and stuff about her sensory things and you think yeah that's totally true like she'll be more awake and looking at you and its true to what it says on the app. and I've share that with family members as well so I'll screenshot it and send it to them and be like 'this is what's happening to Iris'. The white noise app I got a white noisemachine, its upstairs and I thought I'll ge tit on my phone and put it in her moses basket and I did that once and haven't used it again. But sometimes I just use YouTube and play relaxing sounds and put that on the tele in the living room. But I haven't used that app again.

R: So you said you screenshotted something from the Wonder Weeks app, so if this was a digital version is that something you would include?

P: Yeah.

R: So say if you had this digitised, would you want to keep mum and baby stuff separate or would you want it as one thing?

P: You could like, you know like the Ovia app was like 'what's happening to baby this week' and 'what's happenings to mum this week' so it could be two separate little bits. Yeah I suppose maybe separate because I wouldn't share with the rest of the family my stuff or what's happening with me but I would be like here's what's going on with Iris this week and what's happening in her development. I wouldn't be that bothered about going here is me in my mental breakdown this week and here's a photo of it.

R: When you talk about the struggles you had with feeding, did you seek advice from health professionals for that?

P: Yeah the health visitor and the midwives, so both when they visited. Then friends. But it wasn't that helpful, and also breastfeeding support never got in touch and I was told twice that they would get in touch with me and that my number had been passed on. But they never did get in touch so I was waiting for them to get in touch with me but they didn't, so then I tended to just ask friends for advice. But in the end I just went with my gut, because.. I text the health visitor because she had text me and I thought it will be fine but then not helpful really, but then not majorly. I messaged her about upping the teats and I messaged the student midwife, but when I made the decision about switching from breast to bottle I just did it myself. I spoke to friends and family then I just went with my gut, so yeah I didn't seek advice for that but I did about upping the bottle teats, but I don't think I've wrote that yet, it might be week four I can't remember.

R: That's fine. When you mention the sleep struggles on week 2 'Iris is straining a lot and keeping herself awake' did you look for information about that or did you ask anybody about that?

P: Yeah I did I googled quite a lot. I asked the health visitor because I said, that was when she was on breastmilk so at first I thought it was her and the health visitor said it's just while her gut matures and then I googled it loads.. I was like is it.. I didn't really know what the definition of colic was so I googled that and then I was like is it constipation but no she's pooing, so is it trapped wind so I didn't know so I was just googling all the different options. I spoke to the health visitor and she said it's just while her gut matures, then I spoke to some friends because Freddie had bad wind so I spoke to Liz

and Tom and then you mention baby massage so I googled baby massage videos and stuff and did those on her. Then it was when I started realising the bottle of formula I was giving her a day she was settling after, or she wasn't doing it as regularly so that's when I just decided to change.

R: So my next questions are going to be about postpartum. Since giving birth what have been your information needs? So what have you most wanted to find out?

P: Relating to me?

R: Yes relating to yourself.

P: loads about recovery, so like .. about bleeding and how much I should be bleeding because I had days where it was quite a lot so again I text the student midwife, if I didn't have her I would have felt a bit silly ringing the doctors and making an appointment to check so she would just book me in and say yeah come along and get it checked. Stuff about just recovery of things so my stitches and the swelling, and other stuff like postnatal depressing I remember googling that one day because I was feeling low and kept crying and I thought oh god have I got postnatal depression so I googled it and said I think I have, then the next day I was fine and Tom was like you're not depressed. You must have a day when you're like I don't think she likes me or I'm not bonding, then I realise I'd just be having an off day and think oh I'm fine. Stuff like that you can just sit and google is your worst enemy but also you are not given like a little leaflet on things like that are you?

R: No, I didn't get anything.

P: I don't think I got anything on caring for my stitches or caring for the bleeding or even like signs to look out for baby blues, and what to do but nothing so you just sit and look on the internet for it all.

R: Who have you relied on most to provide you information about postpartum care? I know you mentioned using the internet.

P: Internet, definitely my student midwife and I think again friends and my mam and Toms mam just asking people, people going 'I used this bits spray' or 'do these exercises for your coccyx'. Yeah so I thinks it's a mix of women who've had similar things to me and just said what they did so that and the student midwife. She'd come and just check me for stuff.

R: When you had your midwife visits when you came home from hospital, were you able to speak to her about any challenges you were facing about being a new mum?

P: Yes but, Beth the student came and she came with a midwife I had never met before, so I did feel comfortable but it wasn't like, the midwife was really nice but it wasn't anything where I felt like I could have went 'this is happening to me' it was very come look at baby, check the weight, ask if everything's ok, I was breastfeeding when they arrived so I asked them to check if I was feeding right and they looked and said yeah. So it was a bit more surface level, yeah and they weren't there long, 15 minutes I'd say. If you were really struggling with someone or feeling depressed, if I was I wouldn't have felt comfortable saying like I'm a bit down, I don't know what I would have done.

R: Would you more likely, if you were feeling like that to tell friends and family about it?

P: Yeah I think I would tell friends and family and then it would be like, I would wait to see what they say about seeking advice, I don't think I would have went straight to the midwife.

R: Did you feel at that stage when the midwives were visiting your home it was very much focused on baby and less about you?

P: Yeah.

R: So with that, what information have you stored about baby? That could be any information, like little hospital bands or-..

P: Yeah I've got a box with her hospital bands, a box with all her cards off people, I've got an album on my phone of all her pictures, I take loads of pictures each week, then I'll save my favourite pictures into a folder. Other stuff I've got, I suppose it's not information but all her newborn clothes we've put them away, I've got baby record books that I need to fill in. I'd say that was it, in the red book it's got all her measurements in that I want to take a photo of because I didn't even realise there was stuff about the birth. Other than that I'd say that was about it, that little thing from her belly button I've got.

R: Ha yeah I've got Archie's.

P: Yeah it's gross. I think other than that.. letters, she got a bunch of flowers through the door so I cut off the little delivery thing from the front because that was her first with her name and address, and even NHS letters she got one saying she was registered so I kept that little letter, so stuff like that.

R: Where would you store that information and would you share that information with anyone?

P: Probably friends and family and like physical things just little memory boxes I've got, or baby record books.

R: How did you find the transition from when your midwife handed over to your health visitor?

P: Erm, mine was a bit different because Beth the student came out a couple of days later, she came out on day 12, I seen her on day 10 and I forgot my notes, I forgot the folder and she was like I cant properly discharge you without the folder so she said I'll just pop to your house on Monday. Then.. actually I didn't know that they passed you onto the health visitor I just thought it was both of them alongside each other, but fine I suppose. I think it would have been different if I didn't have Beth because I seen her the whole time, so when it was the last one with Beth I was a bit like 'aw'. Then she was like just text me whenever, but I think if it was with the midwife that I hadn't seen that much I wouldn't have been too bothered. Erm.. because the health visitor is really nice.. yeah so not that much of a big deal.

R: Did you find that the information that was provided to you by your midwife and health visitor was different? Or was one better at providing information or gave you more?

P: The health visitor tended to have more information, she had loads of leaflets. So it was like 'this is the baby massage stuff, this is where you're going to register the birth' and forward thinking stuff from the health visitor.

R: Yeah it's more practical isn't it.

P: Yeah I'd definitely say more practical stuff. More about her (baby), I never felt like the health visitor was about me so I never felt comfortable saying oh I'm recovering well or about my stitches, I would talk to the midwife about stuff like that but not her. So say if she came to visit me now and I still had pains and stuff I don't actually know if I would tell her if she asked.

R: Yeah I didn't either.

P: I always just feel like the health visitor is about the baby not about you.

R: I felt awkward talking to my health visitor, she was nice but I didn't feel like it was her job to talk about me. She asked about my mood but I didn't feel like I knew her well enough to say 'I've been feeling a bit anxious' or whatever.

P: Yeah I just keep it surface level about stuff. I didn't feel like I needed to message about the feeding stuff or ask for advice then because again I just felt I didn't really know her. She did text back and she was really nice because I said I was anxious about it and she knew I was on sertraline then she sent a really nice message back. But then again though some information, either she's given me it and I don't know where it is but she sent a message to me saying are you coming to weigh in clinic this week? And I was like am I meant to know the weigh in clinic is this week? So I messaged back and said no when are they? So I only found out when the weigh in clinics were from a text from her and me asking do you know when the weigh in clinics are? I feel like there should just be sheet- I know when the midwife drop ins are because I've used them in pregnancy but I don't know when the weigh in things are and I don't know where little group are, like groups ran by the doctors. I feel like there should be one list of everything you can do.

R: Yeah definitely. I think my health visitor gave me something, with a list of baby groups on. But maybe because mine was Brockwell health visitor so maybe they do it different. She didn't give me a great deal of information it was kind of more about sleep safety and stuff.

P: Yeah she did the sleep safety stuff then asked Tom how he was. Then just sort of went I trust you are a nice couple.

R: Yes I think my health visitor did that.

P: She went 'ee well you's win my couple of the week' I feel very satisfied. Then she was like I'm only going to do the 6 week check, I don't know whether she was going to come back but then she went I'll not bother coming again I'll just come at 6 weeks because she's putting on weight and whatever. But weirdly she told me to keep Otis away when she came, because when she first came Otis was there and she told me to keep him away when she was there because sometimes dogs get protective when they weigh the babies because they cry, so we put him in doggy days, but then when the midwives came we put Otis upstairs and he was barking and the midwife said 'do you not let him out?' and I was like the health visitor said to not let the dog around when you's are here and the midwives were both like what?

R: Oh really?

P: Yeah they were like just let him down, we are in houses where there's about five dogs about, their your family we would never tell you to keep them away so I was like oh right.

R: So I suppose it a bit of conflicting information between the midwife and health visitor?

P: Yeah so I thought if there's any health professionals coming then Otis would have to go away, but then they literally weren't bothered.

R: I used to keep Mila away but only because I didn't want her to jump on the health visitor! So have you used social media to seek advice or information about postpartum related issues? Like forums or anything like that?

P: Forums yes. I joined, but it wasn't that helpful actually- I've been on breastfeeding groups on Facebook and I thought there must be a formula feeding one so I joined a formula feeding one but it's like one from around the world and it's just people talking about different types of formula it's not that useful. But I joined it anyway just to see. Then maybe Instagram I follow a few birth things

and celebrities I like to follow celebrities who've just had a baby and see what their recovery is like. I'd say that's probably about it.

R: Have you watched any YouTube stuff for new mum things or postpartum care?

P: I watch YouTube stuff for babies but I haven't for anything to do with mam stuff, just baby things.

R: So if you were seeking information about postpartum recovery, would you automatically go to an online source?

P: Yeah an online source I think.

R: Would you share information you'd found online with your health visitor or midwife?

P: Yeah.. I wouldn't necessarily screenshot and say this is what I found for this, I would sort of say this is happening and I've read that this could be why. I'd maybe just say I'd done a bit of research or I'd maybe play it off like some of my friends have said this because I know they'd say 'don't trust the internet' and things like that so I would read it then pretend somebody had told me. Yeah I would probably share it with them though.

R: How did you feel when you had to return your pregnancy folder?

P: Like I said last time I didn't realise I had to until you told me, so I was prepared for it. I remember speaking to friends and they were like they take all that away so make sure you take photos of your chart and everything. Then because you get caught up in it so I totally forgot to take any photos of it, so it would have been quite nice to see my bump growth thing, like what was happening each week and every time I met the midwife. Because you sort of forget about you pregnancy journey you just go oh right this is now the baby journey and it would have been nice to reflect back on. So yeah it would have been nice to have some information for a little reflection.

R: Did you feel an emotional connection to that information because it was about you and your journey?

P: Yeah a little bit. You don't have time at the minute to think oh I wish I had that book, but I think once you get into the swing of motherhood and you think about pregnancy you might think oh I wish I had that book. Yeah you do because every time I'd go to a midwife appointment when I was pregnant I'd like looking back through it and seeing what the development was and that was the exciting stuff before you get into motherhood. So yeah I used to like having my little folder.. it's like the first thing you have I suppose , to do with the development and the journey.

R: Yeah definitely. I suppose how would you feel about that being digital and having access to it?

P: Yeah that would be better, it would be better because I kept forgetting it.

R: Oh did you?

P: Yeah I forgot it twice and I had to whizz back home and get it. I think it would be easier to have it online and both of you being able to access it then you can check things. I would leave mine in the car and then I'd think oh I forgot to check like how far down her head was, then I'd think ah it's in the car I'll not bother looking, whereas if it was online you'd be able to screenshot it and send it to people. Especially because I had Liz and Mel who were pregnant as well and I used to say so when you were this many weeks what was your bump size? So it would be nice to just like share that information, but whenever I'd message or she'd message me we'd be like I'll have to check my folder

it's upstairs I'll check later, or like in the car because most time it would be in there. Whereas if it was online it would be readily available.

R: Yes that would be good. Thinking about the red book you have for baby, do you like that being a paper copy?

P: I literally don't do anything with that book. I looked in it yesterday because there's notes slotted in, we haven't registered her birth yet so I've just done it to ring up. So I looked through it to find that bit of paper and I thought I don't know what else is in here and I flicked through it and I realised there's stuff in that I can write in about her like 'my baby is sitting now' and I was like I didn't even know this stuff was in it, the bit in the back and I literally thought, I might be wrong, but I thought it was all for the health professionals so I just hand it over. So I didn't know there was actually bits in for me, but I don't think I would write in it. Like this stuff 'your child's first's and growth charts' so there's stuff there but then there's… like there 'favourite games, finding out about people'.

R: Oh yeah I didn't know that was there.

P: 'Your child's development', it was only because I was flicking through but I wouldn't sit and write in that because I don't think.. I think I'd rather have a nicer little thing.

R: So you think this is information is more for health professionals?

P: Yeah, for them to write in and for me to just check through. I feel like I'd want a nicer book to write in. (looking at red book) see I didn't even know she wrote that in there.

R: I found that with mine too there'd be stuff written in I didn't know about.

P: Maybe she's written stuff and I've not actually looked through it.

R: Do you think if it was digital copy you would use it more?

P: Yeah because it would be easier to go through, I look at this and I think I cant be bothered go through it. Whereas if I could just click on something that said like 'local information' and I'd maybe use it a bit more, but I think this is too big.

R. So almost a bit too much information?

P: Yeah. See like this 'my pregnancy and post-birth well being plan', then I've got no idea what all this about. So you kind of feel like I shouldn't be looking at that but then that is slotted in.

R: So it's kind of two different information, some very medical and personal.

P: Yeah and I feel like there's stuff you shouldn't be looking at. Also I've realised I don't know how long she was when she was born. I tried to have a look where it was written down and I cant find it.

R: I think we got given a sheet from the the hospital.

P: Yeah mine doesn't say on there.

R: So it does seem a mix of information doesn't it because there's sections written by health professionals and medical.

P: Yeah I wouldn't know to find certain things in there.

R: I suppose if it was online it could be under sections which were medical or personal.

P: Yeah like I feel it would take ages sitting looking at the book, like sure start form, I wouldn't even look at this to know that's where they would be. There we go I don't like the red book, get an online version instead!

R: So keeping a journal and things like that, do you feel a personal connection to that because you've written it yourself.

P: Yeah, but actually thinking about it, it would be good to have it as a digital thing, you could do more with it. The picture thing would be really good, you could look at each weeks pictures and think oh she changed so much there, and the little memories from each week would be nice. Then you could have something else physical. I like having a physical thing but I think a digital one would actually be better.

R: So you like the writing process as a therapeutic thing but then to actually store information you'd like the digital version?

P: Yeah.

R: ok, well I think that is all my questions, thank you.

2. Healthcare professional interview schedule

Interview schedule: Midwife

First meeting

Can you describe to me how you approach that first meeting with a client?

- What are the key expectations of that first meeting?
- What are you looking to achieve, what do you need to get done?
    - Medical/ Information gathering
    - Developing the relationship

During the pregnancy

Can you tell me about the general timeline to your meetings antenatal meetings? How many meetings usually take place, what happens at these meetings?

- How would you say your relationship with your client develops over the course of those meetings/the pregnancy?
    - Contact and trust
    - Information gathering
- How do you approach mums who talk about the internet/social media as their sources of information?
- Do you advise mums on any mobile app/websites trusted by health professionals? (baby buddy)
- Can mums contact you outside visits?
  What's the main reason(s) mums to be contact you in this way?

Post-birth and handover

Can you describe to me a typical post birth visit? What kinds of things are important from your perspective?

- Baby focused or mum focused?
- Other stakeholders (dads, other experts)
- A focus on postpartum mental healthcare?

Can you describe to me how you handover to the health visitor at 10-14 days postpartum?

a. What information/concerns are flagged?
b. What do you do if you have concerns or think there may be issues?

Information gathering and exchange

<u>Current: pregnancy folder and red book</u>

When you take back the pregnancy folder with a mums personal information, what is done with this information?

- How do mums tend to feel about the pregnancy folder?

Can you describe how you use the red book with new mums?

What's its value to you and to mum?

Who do you feel the information in pregnancy folder/red book belongs to?

It's being digitised in the next few years – what challenges and opportunities do you see this presenting?

Future:

We are thinking about a mobile app where new mums could write down experiences of motherhood or postpartum symptoms. A recent study we ran involved giving two new mums a journal where they could input this kind of information and any additional struggles or positives they were facing in motherhood.(**Show page from journal and explain what new mums were asked to share)** This might be something that they could share with you or choose to share certain pieces of information. As we hope to design this as a digital version we would like your thoughts on the idea.

Initial reactions:

Positives: value to mums?

What might be shared?

Improvements to sharing/communication? If any? If not, why?

Can you envisage any danger re: this kind of system?

2. Interview schedule- Perinatal women interviews
3. Interview guide- Study 1
4.
5. When you first discovered you were pregnant, what were your information needs?
6. what did you want to find out and where did you go to get that information?
7. Did you download any pregnancy apps at that time to get information from?
8. Did you use any social media/mum forum groups for information needs?
9. If you found information online, were there certain websites that you used?
10. what does pregnancy related information mean to you? (prompts- Is it things like personal stories, facts and figures, what draws you to certain types of information?)
11. Before you got pregnant, when you were trying to conceive if you were, did you do any information searching around that?
12. Did you download any fertility apps or anything then?
13. Do you feel like you did more (information searching) then than you did when you found out you were pregnant?
14. Were there specific dates throughout your pregnancy you feel you can gain access to information,so things that you might have needed, any burning questions you may have had?
15. Do you store any personal information on your apps, or is it generally about baby?
16. What sorts of information do you gain from your midwife, is it more verbal or does she provide you with written information like leaflets?
17. Do you feel like the information you get from your midwife is fulfilling?
18. Do you seek additional information elsewhere? So if your midwife gives you information do you look for it using other sources as well or just accept the information given?
19. Do you feel that the information provided to you has prepared you for pregnancy milestones? This could be things like labour or what to expect at scan dates?
20. Would this mostly be information provided to you by midwife or that you have found yourself?
21. Do you feel you have ample opportunity when you see your midwife to discuss information with her, or symptoms you may have experienced?
22. Do you feel that you've build up a good relationship with your midwife?
23. Which part of information provided to you do you find most useful related to your specific pregnancy and symptoms? As in information provided to you by your health provider.
24. Has your midwife given you any leaflets or any information on how you may feel in pregnancy or anything like that?
25. how do you feel about having access to your own medical notes? The book that you carry round with you through pregnancy?
26. What do you do with this information?
27.
28. do you feel like it is your information and do you feel like you own it?
29. How do think it will feel giving it back when you've kept that information for 9 months?
30. who do you rely on most to provide your health information?
31. how important do you feel your relationship with your midwife, and any other health professional you may have encountered is for during your pregnancy and potentially after your pregnancy too?
32. What is your opinion on accessing online health information?
33. if you found something online, would you ask your midwife to check it?
34. Do you currently use online resources for personal information? And if so which do you use?]

35. Do you use mobile devices such as health apps to gain access to pregnancy information, foetal growth monitoring or symptom management?
36. Do you feel like you value the information more they give you about baby or the information it gives about you?
37. What aspects about the apps do you find most appealing?
38. Do you ever take the information that you've found on those apps and discuss it with your midwife or health provider?
39. Is there anything you would like to see or improve about a website in the way that you access information?
40. if there was an app which you could store your information on it and your midwife could store information it as well and you could use it as a communication tool. Would you use something like that?
41. Do you think it would help improve communication between you and your midwife?

4.Interview schedule- follow-up interviews

1. How have you found using the new mum journal?
2. How do you feel about having a diary which you can store personal information about motherhood/ as opposed to baby record book?
3. Thinking about all types of information, (including photos, websites looked at, written information, diary entries) what have you found most useful relating to yourself?
4. What do you think the benefits of using the new mum journal as a paper copy? (Privacy, ownership of information, personal information)
5. Would you prefer/ use a digital version of the journal? (if not, why?)
6. What information would you want to store in the journal but not able to? (space, time, privacy)
7. Is the information you keep in the journal something you would keep separate from what you share online?
8. Is the information you share via social media more baby focused?
9. Who would you share information from the journal with?

Postpartum information seeking behaviours and needs questions

10. Since giving birth, what have you wanted to find out? About baby and about yourself.
11. Who or what have you relied on most to provide this information?
12. Have you accessed information online about baby/postpartum recovery?
13. Did you discuss any information found online with your midwife/health visitor?
14. Have you used social media to seek advice/information about baby/postpartum related issues?
15. Has the information and support you have received from health professionals been tailored more towards baby or you?
16. When your midwife visited your home, were you able to speak to her about any challenges or worries you were facing about being a new mum?
17. Were you able to discuss mental and physical wellbeing with your midwife?
18. Would you discuss this personal information more with friends/family or health professional?
19. Has the information provided to you by health professionals been written or verbal?
20. What have you taken from the information provided?
21. How have you stored this information?

22. How did you feel when you returned your pregnancy folder?
23. Did you take any copies of the information stored in your folder? If so, which parts?
24. What information have you stored about baby? (photos, hospital letters, hospital bands/baby journal?)
25. Who have you shared this information with? (friends/family, health professionals, social media?)

Interview schedule: Health Visitor

<u>Prior to the first visit</u>

How do you handle the sign-off from midwife to health visitor? What happens?

- Exchange of information
- Cause for concern
- Making contact with a new mum

<u>Planning for the visit? What do you do?</u>

When you initially meet with a mum during pregnancy, how is this visit structured?

- Do you already have information about the mum/family beforehand?
- What are the key expectations of that first meeting?
- What are you looking to achieve, what do you need to get done?
    - Medical/health versus relationship building

<u>Earlier Visits after birth</u>

Information exchange

What information do you provide to a new mum during the first postpartum visit?

- Tailored vs generic

How do you encourage a mum to share personal information with you?

Can you give me an example of an easier home visit and how that compares with a more difficult home visit?

- Explore special circumstances (Multiples/safeguarding issues etc.)
- Who decides how many visits to allocate to each mum? (team meetings, case load, special cases)

<u>Later visits</u>

Can you describe what happens during the last visit with new mum/baby?

- How do you gauge that everything is ok? With mum/with baby?

What do you do if this last visit suggests there are still issues?

- Mum or baby?

Relationships and support

In terms of the key information and other information that you think might be important/relevant to supporting mum and baby…

What kind of support do you/can you offer to a new mum who was **struggling** with postpartum mental health or physical recovery?

During home visits, do you speak to mum alone or with dad/partner present?

- Explore importance of trust and relationship building

How do you approach mums/parents who talk about the internet/social media as their sources of support?

How much of your role is about providing information and support to new mums versus gathering information from mum/baby to monitor baby's health etc.

<u>Red book</u>

Can you describe how you use the red book with new mums?

- What's its value to you and to mum?
- Who do you feel this information belongs to?
- It's being digitised in the next few years – what challenges and opportunities do you see this presenting?

Future:

We are thinking about a mobile app where new mums could write down experiences of motherhood or postpartum symptoms. A recent study we ran involved giving two new mums a journal where they could input this kind of information and any additional struggles or positives they were facing in motherhood. **(Show journal page to explain what information mums shared**). This might be something that they could share with you or choose to share certain pieces of information. As we hope to design this as a digital version we would like your thoughts on the idea.

Initial reactions:

Positives: value to mums?

What might be shared?

Improvements to sharing/communication? If any? If not, why?

Can you envisage any danger re: this kind of system?
